# Effects of Intermittent Fasting on Hypothalamus–Pituitary–Thyroid Axis, Palatable Food Intake, and Body Weight in Stressed Rats

**DOI:** 10.3390/nu15051164

**Published:** 2023-02-25

**Authors:** Cinthia García-Luna, Ixchel Prieto, Paulina Soberanes-Chávez, Elena Alvarez-Salas, Iván Torre-Villalvazo, Gilberto Matamoros-Trejo, Patricia de Gortari

**Affiliations:** 1 Laboratorio de Neurofisiología Molecular, Departamento de Investigaciones en Neurociencias, Instituto Nacional de Psiquiatría Ramón de la Fuente Muñiz, Mexico City 14370, Mexico; 2 Escuela de Dietética y Nutrición, ISSSTE, Mexico City 14070, Mexico; 3 Departamento de Fisiología de la Nutrición, Instituto Nacional de Ciencias Médicas y Nutrición Salvador Zubirán (INCMNSZ), Mexico City 14080, Mexico

**Keywords:** thyroid axis, intermittent fasting, stress, overweight, palatable food

## Abstract

Dietary regimens that are focused on diminishing total caloric intake and restricting palatable food ingestion are the most common strategies for weight control. However, restrictive diet therapies have low adherence rates in obese patients, particularly in stressed individuals. Moreover, food restriction downregulates the hypothalamic–pituitary–thyroid axis (HPT) function, hindering weight loss. Intermittent fasting (IF) has emerged as an option to treat obesity. We compared the effects of IF to an all-day feeding schedule on palatable diet (PD)-stress (S)-induced hyperphagia, HPT axis function, accumbal thyrotropin-releasing hormone (TRH), and dopamine D2 receptor expression in association with adipocyte size and PPARƔ coactivator 1α (PGC1α) and uncoupling protein 1 (UCP1) expression in stressed vs. non-stressed rats. After 5 weeks, S-PD rats showed an increased energy intake and adipocyte size, fewer beige cells, and HPT axis deceleration-associated low PGC1α and UCP1 expression, as well as decreased accumbal TRH and D2 expression. Interestingly, IF reversed those parameters to control values and increased the number of beige adipocytes, UCP1, and PGC1α mRNAs, which may favor a greater energy expenditure and a reduced body weight, even in stressed rats. Our results showed that IF modulated the limbic dopaminergic and TRHergic systems that regulate feeding and HPT axis function, which controls the metabolic rate, supporting this regimen as a suitable non-pharmacologic strategy to treat obesity, even in stressed individuals.

## 1. Introduction

Chronic stress exposure increases the consumption of palatable food in humans and rodents [1,2], leading them to become overweight [3]. A sustained hyperactivation of the adrenal axis maintains an elevated serum concentration of glucocorticoids (GC), which impinges on the hypothalamic arcuate (ARC) neurons, increasing the synthesis of orexigenic peptides, such as neuropeptide Y (NPY), favoring appetite and food-seeking behavior [4].

A high percentage of the obese and hyperphagic population is afflicted with chronic stress [5,6], and, as a common therapeutic strategy, health practitioners routinely recommend a daily food restriction dietary plan that induces a negative energy balance (NEB). Adaptive mechanisms arise in response to NEB, including decreased circulating leptin and insulin levels and a high GC concentration [7]. These peripheral signals downregulate the hypothalamic–pituitary–thyroid (HPT) axis function by decreasing the synthesis of thyrotropin-releasing hormone (TRH) in the hypophysiotropic neurons of the hypothalamic paraventricular nucleus (PVN), which results in low serum levels of thyroid hormones (TH) and a slow degradation of the adipose tissue energy storage [8,9,10]. This adaptive mechanism interferes with the body weight (b.w.) loss of obese patients, which dampens the success rate of this dietary regimen, elevating patient dropout rates from therapeutic programs.

As an alternative to continuous energy restrictive strategies, intermittent fasting (IF) dietary regimens are gaining popularity among patients that need to lose weight [11]. IF, which involves periods of feeding during an animal’s activity phase and periods of voluntary abstinence from food intake, has been successful in improving glycemic control and lipid profile in obese and diabetic patients [12]. Some of the beneficial effects of IF are not due to the loss of weight, but to the metabolic responses to fasting itself [13,14]. The previous findings from our laboratory study show that prepubertal rats with two weeks of IF during the resting phase increase their food intake, their b.w., and present an inhibition of HPT axis activity, which is not observed in rats that are subjected to IF during their activity phase [15].

However, the advantage of IF in reducing hyperphagia in the obese population after being exposed to chronic stress has not been evaluated. Therefore, we aimed to analyze the changes in some parameters of the HPT axis function, such as serum T_3_ levels and the expression of PVN TRH mRNA, in stressed rodents that were subjected to an IF schedule: the time-restricted feeding model, which consists in a daily fixed feeding window of 8 h during the active phase of the rats, with no food restriction [16,17].

The stress and hyperphagia of palatable food are associated with alterations in the accumbal dopaminergic pathway and, specifically, with a low expression of dopamine D2 receptors in the nucleus accumbens (NAc); moreover, the TRH expression in that nucleus is also related to the intake of palatable food in stressed rats [2], however, the effect of an IF schedule on this alteration has not been described. Furthermore, as accumbal TRH participates in the regulation of palatable food intake in chronically stressed rats, we also evaluated its expression levels, as well as those of the D2 receptors in NAc.

We hypothesized that the IF regimen would allow the stressed and the non-stressed animals to lose b.w. when compared to the groups with an all-day schedule, which would be associated with an enhanced HPT axis function and thermogenesis as a result of a high expression of the adipocytes’ mitochondrial proteins. In addition, IF would help the animals to reduce their food intake in association with increased accumbal TRH mRNA levels and D2 expression.

## 2. Materials and Methods

### 2.1. Animals

Male Wistar adult rats (n = 52) with a b.w. of 300 ± 10 g were obtained from the animal facility of the Instituto Nacional de Psiquiatría Ramón de la Fuente Muñiz (INPRFM) and maintained under controlled standard conditions of temperature (24 ± 1 °C) in an inverted light cycle, with lights on from 19:00 to 7:00. The animals were housed in pairs with food and water *ad libitum* for one week of acclimation. All procedures were conducted with the approval of the local Ethics Committee of Animal Experimentation of the INPRFM and following the guidelines outlined in the Mexican Official Norm NOM-062-ZOO-1999.

The rats were randomly assigned to the different experimental groups with regard to stress exposure (housing conditions), type of diet, and feeding schedule.

Stress: The control rats were pair-housed (non-stressed) (C, n = 26) or single-housed (isolation-stressed, S, n = 26). Isolation housing is a non-invasive model of chronic stress that induces GCs serum chronic elevation and promotes the hyperphagia of standard chow and palatable diet, as well as an increase in body weight [18,19].

**Figure 1:**
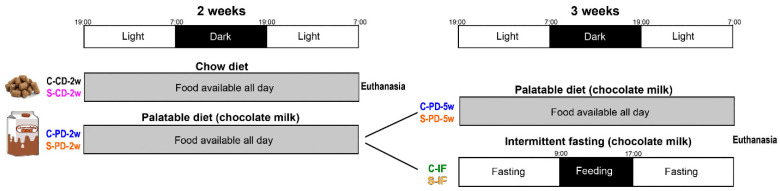
Experimental design. Feeding schedule of control (C, pair-housed) and stressed (S, single-housed) adult male rats. C and S rats had *ad libitum* access to chow (CD) or palatable diet (PD, chocolate milk) for 2 weeks. Once that time elapsed, all CD-fed and half of the PD-fed animals (C-CD-2w, S-CD-2w, C-PD-2w, and S-PD-2w) were euthanized. The remaining C-PD and S-PD rats were divided and subjected to two different feeding schedules: intermittent fasting (IF: feeding window of 8 h (9:00–17:00) during the activity phase: C-IF, S-IF) or fed all day for 3 more weeks. Rats fed all day with a PD had a cumulative intake time of 5 weeks (C-PD-5w, S-PD-5w). At the end of the 5th week, all animals were euthanized.

Diet: The rats from each housing group were divided into the following two subgroups: a control diet (CD) group that was fed regular chow (5001, PMI Nutrition International, Brentwood, CA, USA, energy = 3.35 Kcal/g, protein = 28.8%, fat = 13.4%, carbohydrates = 56.7%) and a palatable diet (PD) group that was fed regular chow and an energy-dense beverage that consisted of chocolate milk prepared with lactose-free whole cow milk (Alpura, Ecatepec, Mexico) and chocolate powder (Nesquik, Nestlé, Tlalnepantla, Mexico; 5.75 g/100 mL; energy = 0.76 Kcal/mL, protein = 16.1%, fat = 37.6%, carbohydrates = 46%) for 2 weeks: C-CD-2w (n = 6), S-CD-2w (n = 6), C-PD-2w = (n = 20), and S-PD-2w (n = 20). Pair-housed rats were offered two bottles of PD and single-housed animals were offered one bottle of PD. The chow pellets were pre-weighed and offered freely on a daily basis and the chocolate milk was prepared daily and was available *ad libitum*; both foods were offered 2 h after the onset of the dark phase (9:00). All groups had unlimited access to tap water. The b.w. and food intake (cumulative over 24 h) were quantified daily between 8:00 and 10:00 over a 2-week period. Chow food and chocolate milk intakes were calculated by subtracting the amount of food offered minus the amount weighed on the next day from the initial amount. After 2 weeks, the C-CD-2w and S-CD-2w animals (n = 6/group) and the C-PD-2w and S-PD-2w rats (n = 4/group) were euthanized (Figure 1). Trunk blood was collected and centrifuged at 3000 rpm for 30 min at 4 °C to separate serum; brain and mesenteric adipose tissue were rapidly dissected, frozen, and maintained at −70 °C for mRNA and protein abundance analyses. Another adipose tissue sample from each rat was fixed in formalin for histological analysis.

Feeding schedule: The remaining C and S rats that were fed with a PD for 2 weeks (n = 16/group) were divided into 2 groups subjected to 2 different feeding schedules. The rats were maintained either with food available all day (PD) (C-PD, S-PD; n = 8/group) or with an intermittent fasting regimen (IF) (C-IF, S-IF; n = 8/group) for 3 additional weeks. Rats fed all day with a PD had a cumulative intake time of 5 weeks (C-PD-5w, S-PD-5w). IF consisted of a feeding window of 8 h (9:00–17:00) during the activity phase. B.w. and regular chow and chocolate milk intake were registered daily, as described above. At the end of the 5th week, all animals were euthanized as described below (Figure 1). Trunk blood and brain and mesenteric adipose tissue were collected.

### 2.2. Brain and Adipose Tissue mRNA Quantification by Real-Time PCR Analysis

Coronal slices from frozen NAc (3.00 mm to 0.6 mm from bregma), PVN (−1.08 mm to −2.16 mm from bregma), and ARC (−2.16 mm to −3.36 mm from bregma) [20] were punch-dissected employing a 1 mm diameter sample corer. The total RNA of each region, and from adipose tissue, was isolated using the guanidinium thiocyanate–phenol–chloroform isoamyl alcohol technique, as described [21]. Briefly, guanidinium thiocyanate reagent was added to frozen tissue samples, followed by homogenization with a sonicator. Phenol–chloroform isoamyl alcohol (3/10 of guanidinium thiocyanate reagent volume) was added to the homogenate, incubated for 15 min, and centrifuged at 10,000 rpm for 20 min at 4 °C. The aqueous phase was transferred to another tube and mixed with isopropanol (1:1 of aqueous phase volume). After overnight incubation at −20 °C, the samples were centrifuged at 13,800 rpm for 30 min at 4 °C, followed by two washes with cold 75% ethanol. The pellet was vacuum-dried and dissolved in DEPC-treated water. The RNA concentration was determined using a Biophotometer (Eppendorf, Hamburg, Germany) and the integrity was assessed with 1% agarose gel electrophoresis using ethidium bromide as a marker and visualized with a UV transilluminator. The optical density of the bands was analyzed with ImageJ software (NIH, Washington, MA, USA). RNA (1.5 μg) was used for complementary DNA (cDNA) synthesis with M-MLV reverse transcriptase (Invitrogen, ThermoFisher Scientific, Waltham, MA, USA). Quantitative PCR was performed with TaqMan Gene Expression probes and TaqMan Universal PCR Master Mix (ThermoFisher Scientific, Waltham, MA, USA) in a StepOnePlus Real-Time PCR System (ThermoFisher Scientific, Waltham, MA, USA). The TaqMan gene expression probes were as follows: *Actb*, Rn00667869_m1; *Trh*, Rn00564880_m1; *Npy*, Rn01410145_m1; *Ucp1*, Rn00562126_m1; and *Ppargc1a*, Rn00580241_m1. The mRNA levels of all genes were normalized using actin b as a housekeeping gene. Each sample was run in duplicate. The amplification program was 40 cycles of 95 °C for 15 s, then 60 °C for 1 min. The mRNA levels were quantified via the ΔΔCt method and the percentage of change in the gene of interest for each group was obtained using the following equation: ΔΔCt = 2 − e(ΔCt = Ct Control − ΔCt experimental group) × 100.

### 2.3. Determination of Serum Hormones Content

The serum CORT concentration was measured with a competitive ELISA kit (Enzo Life Sciences Inc., New York, NY, USA), following the manufacturer’s instructions (sensitivity: 27 pg/mL, and 8.4% and 8.2% of inter- and intra-assay variation, respectively).

The total T_3_ serum content was determined using a commercial ELISA kit (Alpco, Salem, NH, USA), according to manufacturer’s instructions. This product has a coefficient of variation for inter-assay of 3.2%, an intra-assay of 4.1%, and a sensitivity of 0.2 ng/mL.

### 2.4. Immunoblotting

The right NAc was used to determine D2 receptor expression. A total of 100 µL of RIPA lysis buffer per tissue sample (Abcam, Cambridge, UK) with protease inhibitor (1:100, Thermo Fisher Scientific, Waltham, MA, USA) was used to extract proteins using a sonicator. Tissue lysates were incubated for 20 min on ice and then centrifuged at 13,800 rpm at 4 °C for 10 min. Supernatants were collected, the same volume of 2X Laemmli buffer (Sigma-Aldrich, St. Louis, MO, USA) was added, and the samples were denatured at 95 °C for 5 min. A total of 1 µL aliquot was used for protein determination by the micro-Lowry method.

Protein samples (30 µg) were loaded in 10% SDS-PAGE gels for electrophoresis and, after 1 h, were transferred to Hybond-C extra nitrocellulose membranes (Amersham, LifeScience, Buckinghamshire, UK). The membranes were then incubated in a blocking solution of 5% BSA in 1X PBS/Tween 0.05% for 1 h and for 72 h with the primary antibody for D2 receptors (1:1000, rabbit anti-D2, AB5084P, Merck Millipore, Darmstadt, Germany) in the blocking solution. After washing with PBS/0.1% Tween, the membranes were incubated for 1 h with the secondary antibody (1:10,000 goat anti-rabbit HRP conjugate; 6721 Abcam, Cambridge, UK) in 3% BSA. The membranes were then incubated with the polyclonal goat anti-actin antibody (1:1000, sc-1616, Santa Cruz Biotechnology, Inc., Dallas, TX, USA) as a loading control. Protein bands were revealed using luminol and visualized with a densitometer iBright CL1000 imaging system (Invitrogen, Waltham, MA, USA). ImageJ software was used for the analysis and quantification of luminescent signals and the values are the intensity of the D2/actin signals in arbitrary units.

### 2.5. Adipose Tissue Histologic Analysis and Immunohistochemistry (IHC)

Mesenteric adipose tissue samples were fixed in PBS-buffered 4% paraformaldehyde, dehydrated, embedded in paraffin, and cut into pairs of 4 µm sections. For adipocyte morphology analysis, one section was stained with hematoxylin and eosin (H&E) and digitized in a Leica DM750 microscope (Leica, Wetzlar, Germany) using a 20X lens. Analysis of the adipocyte areas was performed using Adiposoft software, as previously described [22]. The adaptive thermogenesis marker UCP1 was determined in the other obtained tissue section, as described previously [23]. Briefly, endogenous peroxidase was blocked with 3% H_2_O_2_ solution. Then, non-specific background staining was avoided by using the immunohistochemistry background blocker (Enzo Life Sciences, New York, NY, USA). The adipose tissues were incubated with rabbit monoclonal anti-mouse UCP1 antibody (Abcam, Cambridge, UK) diluted at 10 µg/mL for 40 min at room temperature. Binding was identified with Universal Dako-labeled streptavidin–biotin secondary antibody (Dako, Glostrup, Denmark). The slides were incubated with streptavidin peroxidase for 15 min, followed by incubation with the peroxidase substrate 3,3′-diaminobenzidine (DAB; Sigma-Aldrich, St. Louis, MO, USA) for 10 min. The sections were counterstained with hematoxylin, dehydrated with alcohol and xylene, and mounted in resin. The negative controls were incubated with an IHC universal negative control reagent (IHC universal negative control reagent, Enzo Life Sciences, Inc., New York, NY, USA) instead of a primary antibody. Digital images were taken of each section at 40X magnification and the staining areas were quantified with ImageJ software.

### 2.6. Statistical Analysis

In order to have independent observations and to compare data collected under two different housing conditions (grouped, no stress vs. isolation, stress) within the same analysis, we used the amount of food eaten per cage of the pair-housed animals as the experimental unit. Thus, for the single-housed animals, we added the amount of food eaten by two rats by randomly selecting the pair of rat’s food intake to be added with an algorithm.

B.w. gain and energy intake were analyzed by two-way repeated measures ANOVA (stress × diet along time; stress × feeding schedule along time). The adipose tissue parameters’ comparisons between C-PD-2w and S-PD-2w were analyzed using Student’s *t*-tests. The gene (TRH, NPY, PPARƔ coactivator 1α (PGC1α), and uncoupling protein 1 (UCP1)) expression, hormone levels (CORT and T_3_), adipocyte size, number of beige adipocytes, and accumbal D2 receptor content were analyzed using a two-way ANOVA (stress × type of diet; stress × feeding schedule). When *p* was <0.05, Bonferroni’s post hoc test was performed.

## 3. Results

### 3.1. Changes after 2 Weeks in Stressed and Non-Stressed Rats Eating Chow or Palatable Food

#### 3.1.1. Body Weight Gain

**Figure 2:**
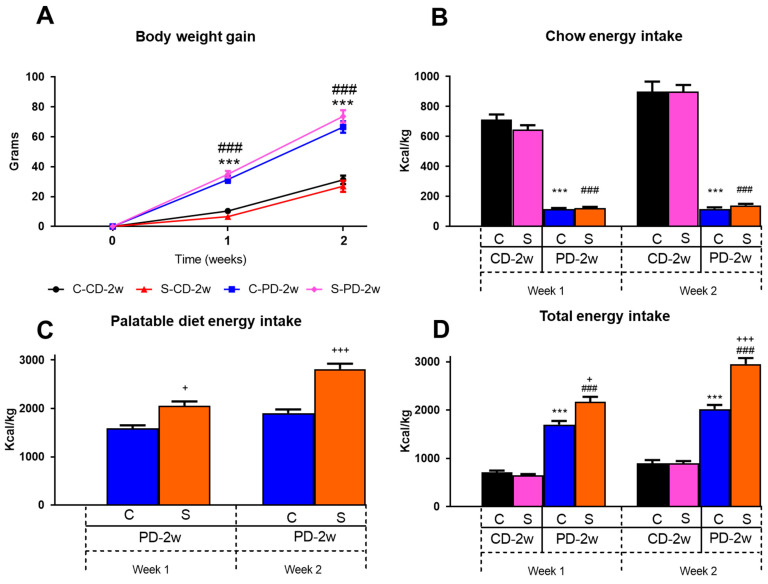
Effect of stress and a palatable diet on body weight and energy intake. (**A**) Body weight (b.w.) gain of the control (C) or stressed (S) rats fed a regular chow diet (CD) or palatable diet (PD, chocolate milk ingestion) for 2 weeks (C-CD-2w, S-CD-2w, C-PD-2w, and S-PD-2w), n = 6 rats/group in C-CD-2w and S-CD-2w; n = 20 rats/group in C-PD-2w and S-PD-2w. (**B**) Chow energy intake, (**C**) Palatable diet energy intake, and (**D**) Total energy intake (Kcal/Kg b.w.), n = 3 cages/group in C-CD-2w and S-CD-2w; n = 10 cages/group in C-PD-2w and S-PD-2w. Values are the mean ± SEM. *** *p* < 0.001 vs. C-CD-2w; ^###^ *p* < 0.001 vs. S-CD-2w, ^+^ *p* < 0.05, ^+++^ *p* < 0.001 vs. C-PD-2w.

On both of the experimental weeks, the b.w. gain between the non-stressed C-CD-2w and the isolation-stressed S-CD-2w rats, as well as between the C-PD-2w and S-PD-2w groups, was similar. In contrast, after the 1st week of providing the animals with a PD containing chocolate milk, the C-PD-2w and S-PD-2w animals had a 3-fold increase in b.w. gain when compared to their respective chow-fed groups (C-CD-2w = 100%, 10.4 ± 1.5 g of b.w. gain; S-CD-2w = 6.6 ± 1.4 g of b.w. gain). In the 2nd week, the b.w. gain of both the C-PD-2w and the S-PD-2w groups was 2-fold higher than that of their respective chow-fed controls (C-CD-2w = 100%, 31.2 ± 2.6 g of b.w. gain; S-CD-2w = 27 ± 3.4 g of b.w. gain). The two-way repeated measures ANOVA of b.w. gain showed an effect of diet (palatable vs. chow) (F_(1,147)_ = 334.38, *p* < 0.001) and time (F_(2,147)_ = 195.80, *p* < 0.001), but there were no differences between the stress conditions (paired vs. single-grouped) (Figure 2A). Those results revealed that consuming the palatable diet increased b.w. gain, but that stress did not affect this parameter.

#### 3.1.2. Energy Intake

The amount of energy ingested from chow pellets between the stressed and the control groups was similar throughout the experiment, revealing that stress alone did not impact the consumption of the regular food (Figure 2B). However, when the animals had access to a PD, in the first week C-PD-2w showed a reduction of 84% in their energy intake from chow in comparison to that of the animals that were consuming only chow (C-CD-2w) (100%; 712 ± 35 Kcal/Kg); also, a reduction of 81% in the S-PD-2w rats vs. the S-CD-2w group (647 ± 28 Kcal/Kg) was found (Figure 2B). On the 2nd week, the reduction was 87% in the C-PD-2w rats and 84% in the S-PD-2w rats vs. their chow-fed controls (C-CD-2w = 100%: 899 ± 66 Kcal/Kg; S-CD-2w = 899 ± 45 Kcal/Kg) (Figure 2B).

Regarding the intake of a PD, isolation stress had an effect on the amount of chocolate milk consumed, given that in the 1st week S-PD-2w increased its PD energy intake by 129%, and on the 2nd week it raised to 148% in comparison to that of the C-PD-2w animals (100%, week 1, 1587 ± 66 Kcal/Kg, week two 1906 ± 75 Kcal/Kg) (Figure 2C). Additionally, regarding the combined intake of chow and PD, both the control C-PD-2w and the stressed S-PD-2w animals in the 1st week increased their total energy intake to 239% and 336% vs. that of C-CD-2w (100%; 712 ± 35 Kcal/Kg) and of S-CD-2w (647 ± 28 Kcal/Kg), respectively, due to the intake of chocolate milk (Figure 2D). This trend was maintained during the 2nd week, suggesting that the animals preferred to consume a PD independently of being exposed or not to stressful conditions and despite the availability of regular chow food. Two-way repeated measures ANOVA showed a significant effect of diet (PD vs. CD): F_(1,66)_ = 268.54, *p* < 0.001, time: F_(2,66)_ = 7.2, *p* < 0.05, and stress conditions: F_(1,66)_ = 8.89, *p* < 0.01, and an interaction between those variables: F_(4,66)_ = 2.86, *p* < 0.05. This observation corroborated that stress is a factor that exacerbates the consumption of a highly palatable diet, resulting in an increase in total energy intake.

#### 3.1.3. Adipose Tissue Morphology and Thermogenesis

Table 1 shows that, after 2 weeks, stress increased the adipocytes’ size in the mesenteric adipose tissue of the PD-fed animals by 37% vs. that of the C-PD-2w group. The Student’s *t*-test showed a significant difference between the groups (t_(1243)_ = 7.51, *p* < 0.001). It was also observed that the number of beige adipocytes, as well as the mRNA levels of UCP1 and PGC1α, decreased after 2 weeks to 36%, 47%, and 23%, respectively, vs. C-PD-2w. The effect was significant for the beige adipocytes (t_(16)_ = 1.923, *p* < 0.05; UCP1 t_(8)_ = 2.165, *p* < 0.05, and PGC1α t_(8)_ = 4.045, *p* < 0.01).

#### 3.1.4. Neuroendocrine Changes

**Figure 3:**
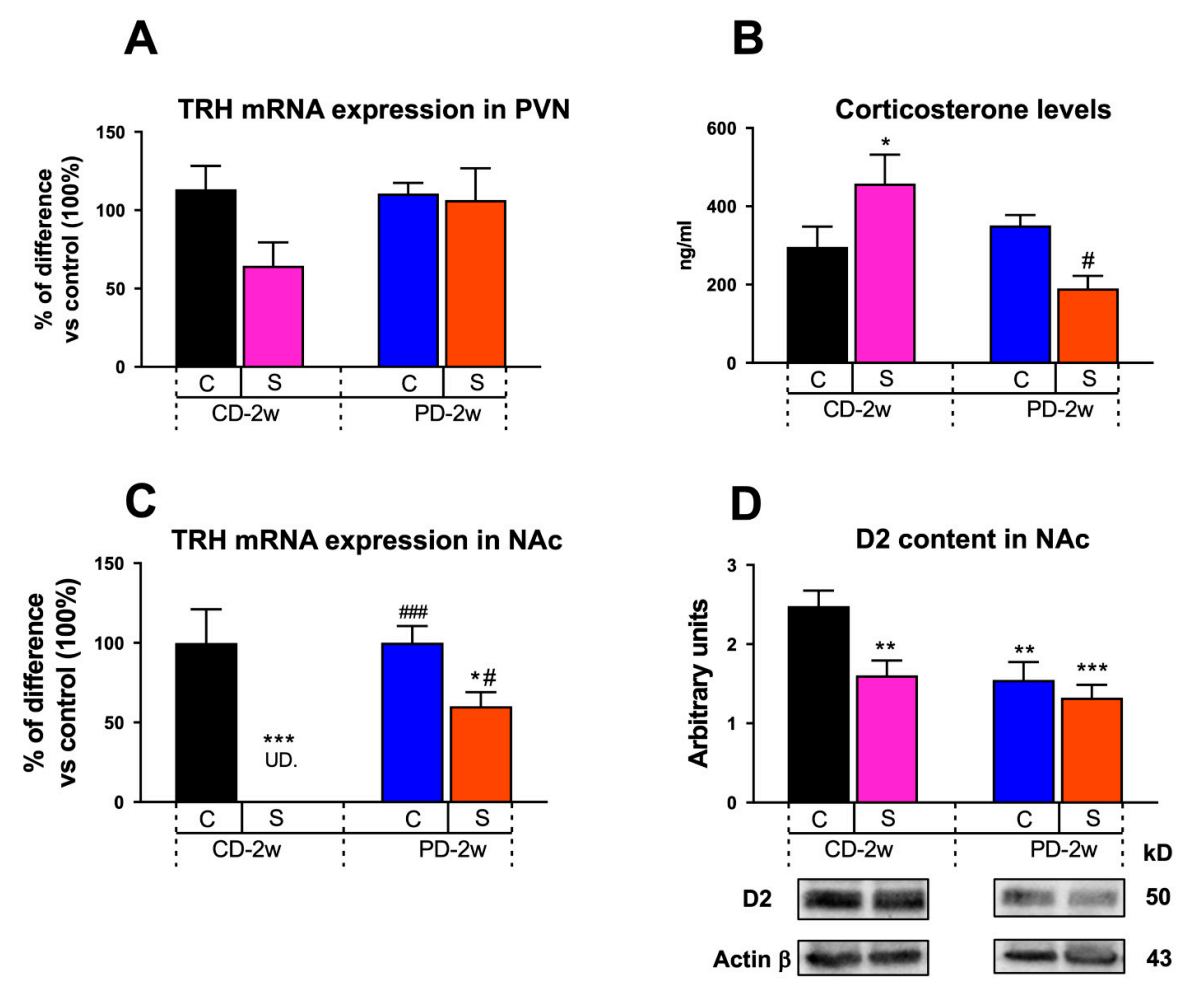
Effect of stress and palatable diet intake on neuroendocrine and limbic parameters. (**A**) TRH mRNA expression in the hypothalamic paraventricular nucleus (PVN), (n = 3–4 rats/group). (**B**) Serum corticosterone levels, (n = 3–6 rats/group). (**C**) TRH mRNA expression in the nucleus accumbens (NAc), (n = 3–4 rats/group). (**D**) Dopamine receptor type 2 (D2) content in the NAc., (n = 3–4 rats/group). Data represent the mean ± SEM of mRNA levels expressed as arbitrary units (**A**,**C**), ng/mL (**B**), and optical density arbitrary units (**D**). U.D.: undetectable. * *p* < 0.05, ** *p* < 0.01, *** *p* < 0.001 vs. C-CD-2w; ^#^ *p* < 0.05, ^###^ *p* < 0.001 vs. S-CD-2w.

This study investigated whether stress alone or combined with palatable food intake changed PVN TRH mRNA expression after 2 weeks. Here, we observed only a tendency to decrease the TRH mRNA levels in S-CD-2w vs. C-CD-2w (100%, 1.1 ± 0.2 a.u.; Figure 3A), which would support the described effect of high CORT levels (Figure 3B) on down regulating the peptide’s expression [24].

Figure 3B shows that, as expected, the CORT serum levels of the S-CD-2w group increased to 185% vs. C-CD-2w (100%, 267 ± 43 ng/mL). Importantly, when the stressed rats (S-PD-2w) ingested chocolate milk, their CORT levels decreased to 38% vs. S-CD-2w (495 ± 58 ng/mL). Two-way ANOVA showed an effect of the dietary regimen of F_(1,13)_ = 7.072, *p* < 0.05, stress of F_(1,13)_ = 14, *p* < 0.05, and a significant interaction between the variables of F_(1,13)_ = 14.65, *p* < 0.05.

#### 3.1.5. Limbic Changes

Stress exposure reduced the accumbal TRH peptide expression to undetectable levels in the S-CD-2w animals vs. the controls, whereas the TRH mRNA levels in the NAc of S-PD-2w increased vs. those of S-CD-2w but did not reach the levels of the C-PD-2w group (Figure 3C). Two-way ANOVA showed an effect of stress of F_(1,17)_ = 34.22, *p* < 0.001, feeding schedule of F_(1,17)_ = 13.77, *p* < 0.05, and a significant interaction between the variables of F_(1, 17)_ = 6.33, *p* < 0.05.

After two weeks, the stressed groups, either chow- or PD-fed, showed a similar reduction to 65% and 58%, respectively, in D2 content in NAc vs. C-CD-2w (100%, 2.5 ± 0.2 a.u.); additionally, the PD intake by itself in the non-stressed animals (C-PD-2w) induced the same decrease (to 69%) as that of S-CD-2w and of S-PD-2w. No additive effect of the PD and stress was observed (Figure 3D). Two-way ANOVA showed a significant effect of stress of F_(1,12)_ = 8.11, *p* < 0.05 and feeding schedule of F_(1,12)_ = 9.84, *p* < 0.01.

### 3.2. Changes Induced by Three Weeks of Intermittent Fasting in Stressed and Non-Stressed Rats Eating Palatable Food

#### 3.2.1. Body Weight

**Figure 4:**
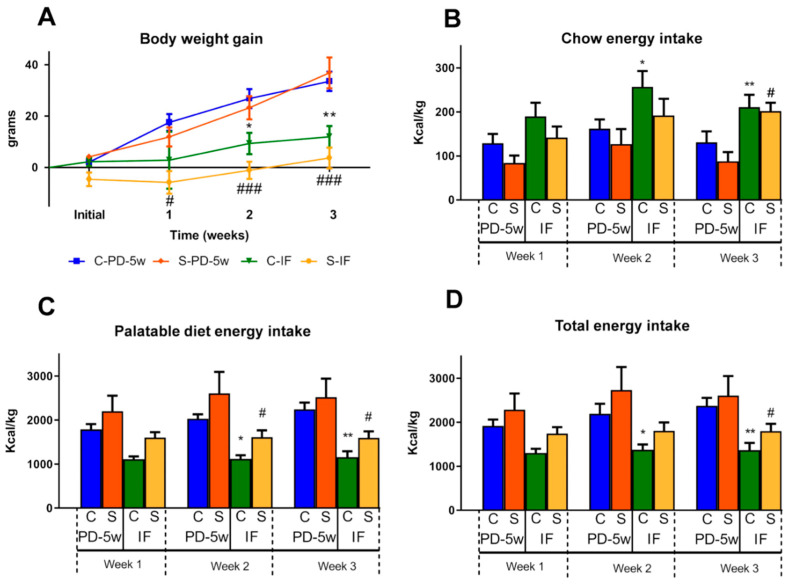
Effect of stress and intermittent fasting on rats fed with a palatable diet. (**A**) Body weight gain of the control or stressed animals fed with a PD and subjected to two feeding schedules: food available all day (with a cumulative intake time of 5 weeks, C-PD-5w, S-PD-5w) or intermittent fasting (C-IF, S-IF) for 3 additional weeks. Data are represented as the mean ± SEM of grams of b.w. (n = 8 rats/group); (**B**) Chow energy intake; (**C**) Palatable diet energy intake; and (**D**) Total energy intake (Kcal/Kg b.w; n = 4 cages/group. Data represent the mean ± SEM. * *p* < 0.05, ** *p* < 0.01 vs. C-PD-5w; ^#^ *p* < 0.05, ^###^ *p* < 0.001 vs. S-PD-5w.

After two weeks of isolation stress and eating a PD, the C and S animals were subdivided into two groups and maintained for three more weeks with all-day feeding or IF schedules (with a feeding window of 8 h during the activity phase). We observed that the stressed rats with continuous access to palatable food (S-PD-5w) did not have a different b.w. gain compared to the C-PD-5w group at any week of the experiment. However, both of the groups with intermittent fasting (C-IF, S-IF) showed a lower b.w. gain than their respective all-day-fed controls (C-PD-5w and S-PD-5w), but with no difference between them (Figure 4A).

Regarding the effect of IF, in the 2nd week the C-IF group reduced their b.w. gain to 35%, and in the 3rd to 36% of that of C-PD-5w (100%, 27 ± 4 g in week two; and 34 ± 4 g in week three). S-IF reduced 105% of their b.w. gain on week two and decreased to 90% on week three vs. S-PD-5w (100%, week two: 23 ± 5 g b.w. gain; week three: 37 ± 6 g). Two-way repeated measures ANOVA showed an effect of stress of F_(1,83)_ = 4.05, *p* < 0.05, time of F_(2,83)_ = 7.04, *p* < 0.001, and feeding schedule of F_(1,83)_ = 52.96, *p* < 0.001 (Figure 4A).

#### 3.2.2. Energy Intake

When the animals were fed all day, stress exposure (S-PD-5w) did not induce a greater ingestion of calories from the chow, from the PD, or from the total energy intake when compared to that of C-PD-5w at any week that was analyzed (Figure 4B–D). C-IF increased the energy intake from the chow pellets to 159% on week two and to 160% on week three, when compared to C-PD-5w (100%, week two: 162 ± 21; week three: 131 ± 25 Kcal/Kg), and S-IF increased the chow energy intake until week three to 229% vs. S-PD-5w (100%, 88 ± 21 Kcal/Kg) (Figure 4B). Interestingly, the C-IF animals also decreased their PD ingestion to 54% after two weeks, and to 52% on week three vs. C-PD-5w (100%, week two: 2330 ± 96; week three: 2243 ± 154 Kcal/Kg) (Figure 4C), and S-IF reduced their palatable food intake to 60% after two weeks and to 63% on week two vs. that of S-PD-5w (100%, week two: 2997 ± 556; week three: 2518 ± 427 Kcal/Kg) (Figure 4C), but there was no difference between C-IF and S-IF (Figure 4C).

Considering the total energy intake, C-IF showed a significant reduction to 60% on week two and to 58% on week three in comparison to C-PD-5w (100%, week two: 2492 ± 90; week three: 2374 ± 140 Kcal/kg). When compared to S-PD-5w (100%, week three: 2606 ± 449 Kcal/kg), S-IF also reduced their total energy intake to 69% after three weeks (Figure 4D).

Two-way repeated measures ANOVA showed an effect of stress of F_(1,51)_ = 9.36, *p* < 0.01; time of F_(3,51)_ = 5.91, *p* < 0.05, feeding schedule of F_(1,51)_ = 37.35, *p* < 0.0001, and an interaction between time and feeding schedule of F_(3,51)_ = 5.07, *p* < 0.01.

#### 3.2.3. Adipose Tissue Morphology and Thermogenesis

**Figure 5:**
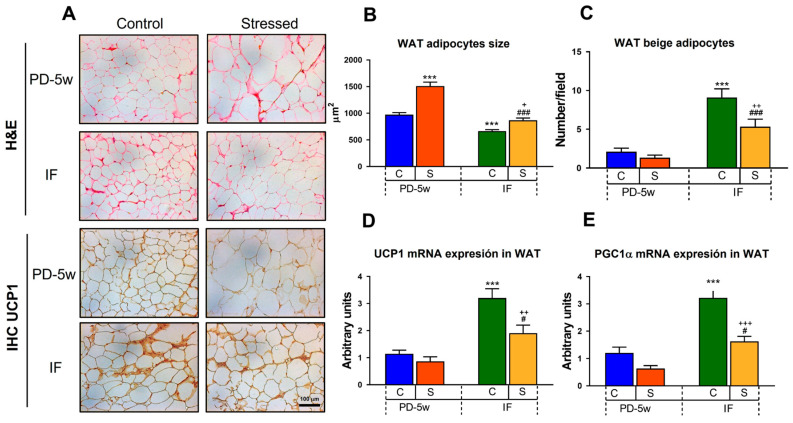
Effect of stress and intermittent fasting on adipose tissue morphology and thermogenesis parameters. (**A**) Representative photomicrographs of hematoxylin and eosin (H&E) and UCP1 immunostaining of mesenteric white adipose tissue (WAT) at 20X magnification of the control or stressed animals fed with a PD and subjected to two feeding schedules: food available all day (with a cumulative intake time of 5 weeks, C-PD-5w, S-PD-5w) or intermittent fasting (C-IF, S-IF) for 3 additional weeks. Scale bar 100 µm. (**B**) Mesenteric adipocytes’ size. Data are represented as the mean ± SEM of µm^2^. (**C**) Number of beige adipocytes per field analyzed. Data are represented as the mean ± SEM of the total number of adipocytes/field. (**D**) UCP1 and (**E**) PGC1α mRNA expression in the WAT. Values are the mean ± SEM of the relative abundance of mRNA levels expressed in arbitrary units. n = 4 rats/group. *** *p* < 0.001 vs. C-PD-5w; ^#^ *p* < 0.05, ^###^ *p* < 0.001 vs. S-PD-5w; ^+^ *p* < 0.05, ^++^ *p* < 0.01, ^+++^ *p* < 0.0001 vs. C-IF.

Hematoxylin and eosin staining of the mesenteric adipose tissue sections (Figure 5A) revealed that isolation stress increased the mean size of the adipocytes in S-PD-5w to 155% when compared to that of C-PD-5w (100%, 973.5 ± 37 µm^2^), indicating impaired adipogenesis leading to hypertrophic growth (Figure 5B). Interestingly, the IF regimen was able to decrease the adipocyte size in both C-IF and S-IF to 68% and 57% of that of C-PD-5w (fed all day) or S-PD-5w, respectively (Figure 5B). For the adipocyte sizes, two-way ANOVA showed an effect of stress exposure of F_(1,842)_ = 47.459, *p* < 0.001, feeding schedule of F_(1,842)_ = 79.25, *p* < 0.001, and an interaction between the variables of F_(1,842)_ = 9.894, *p* < 0.01.

IHC UCP1 staining (Figure 5A) showed that IF increased the number of beige adipocytes to 431% in C-IF and to 400% in S-IF vs. their respective all-day-fed groups (C-PD-5w = 2 ± 0.5 cells/field; S-PD-5w = 1 ± 0.3 cells/field) (Figure 5C). To confirm the IHC findings, we evaluated the mRNA expression of UCP1 and of the PGC1α in the adipose tissue. We observed that IF increased the UCP1 mRNA levels to 279% in C-IF and to 220% in S-IF groups vs. C-PD-5w and S-PD-5w, respectively, (C-PD-5w = 100%, 1.76 ± 0.2 a.u.; S-PD-5w = 100% 1.32 ± 0.3 a.u.) (Figure 5D), and the PGC1α mRNA content to 266% and 256% in C-IF and S-IF rats vs. their respective all-day-fed groups (C-PD-5w = 100%, 1.03 ± 0.2 a.u.; S-PD-5w = 100%, 0.54 ± 0.1) (Figure 5E). Two-way ANOVA showed an effect of stress of F_(1,32)_ = 8.311, *p* < 0.01 and feeding schedule of F_(1,32)_ = 48.46, *p* < 0.001 for brite adipocytes; an effect of stress of F_(1,16)_ = 9.695, *p* < 0.01 and feeding schedule of F_(1,16)_ = 36.766, *p* < 0.001 for the UCP1 mRNA levels; and an effect of stress of F_(1,16)_ = 16.344, *p* < 0.01 and feeding schedule of F_(1,16)_ = 31.167, *p* < 0.001 for the PGC1α mRNA content.

#### 3.2.4. Neuroendocrine Changes

**Figure 6:**
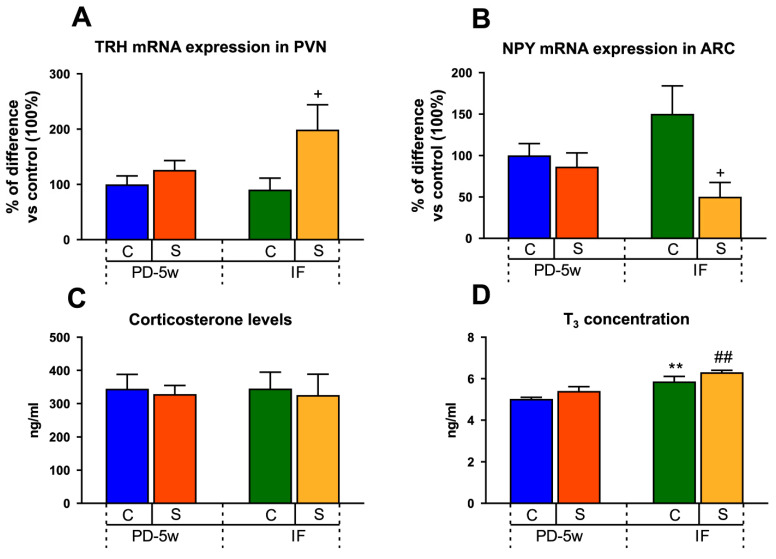
Effect of stress and intermittent fasting on neuroendocrine parameters. (**A**) TRH mRNA expression in the hypothalamic paraventricular nucleus (PVN) and (**B**) NPY mRNA expression in the hypothalamic arcuate nucleus (ARC) of the control or stressed animals fed with a PD and subjected to two feeding schedules: food available all day (with a cumulative intake time of 5 weeks, C-PD-5w, S-PD-5w) or intermittent fasting (C-IF, S-IF) for 3 additional weeks. Values are the mean ± SEM of the mRNA levels expressed in % of difference vs. C-PD-5w (n = 4 rats/group). (**C**) Corticosterone and (**D**) T_3_ serum levels expressed as ng/mL. Data are the mean ± SEM (n = 4 rats/group). ** *p* < 0.01 vs. C-PD-5w; ^##^ *p* < 0.01 vs. S-PD-5w; ^+^ *p* < 0.05 vs. C-IF.

The S-IF rats showed a significant upregulation of PVN TRH mRNA expression by 119% when compared to C-IF (100%, 2 ± 0.9 a.u.) (Figure 6A), but it was similar to S-PD-5w. Two-way ANOVA revealed an effect of feeding schedule of F_(1,12)_ = 9.235, *p* < 0.01 and stress exposure of F_(1,12)_ = 3.683, *p* < 0.05.

The ARC NPY mRNA content decreased only in the S-IF group to 59% when compared to C-IF (100%, 1.02 ± 0.15 a.u.) (Figure 6B). Two-way ANOVA showed an effect of stress and feeding schedule of F_(1,11)_ = 3.969, *p* <0.05.

The CORT values in S-PD-5w were similar to those of C-PD-5w. Knowing that low food intake is a stressor, it was interesting to observe that the C-IF group did not show an increase in CORT levels, even when that group ate less energy than C-PD-5w; more importantly, the combination of reduced food intake and chronic stress exposure in the S-IF rats was unable to increase the CORT levels (Figure 6C).

When analyzing the T_3_ levels, we observed that the rats under an IF schedule, either with or without stress exposure (S-IF, C-IF), showed 16% more hormone serum concentration than that of the groups with access to food all day (C-PD-5w: 5 ± 0.03, S-PD-5w: 5.4 ± 0.2 ng/mL) (Figure 6D), which could have facilitated their reduced b.w. gain. Two-way ANOVA showed differences in the feeding schedule of F_(1,27)_ = 26.336, *p* < 0.001.

#### 3.2.5. Limbic Changes

**Figure 7:**
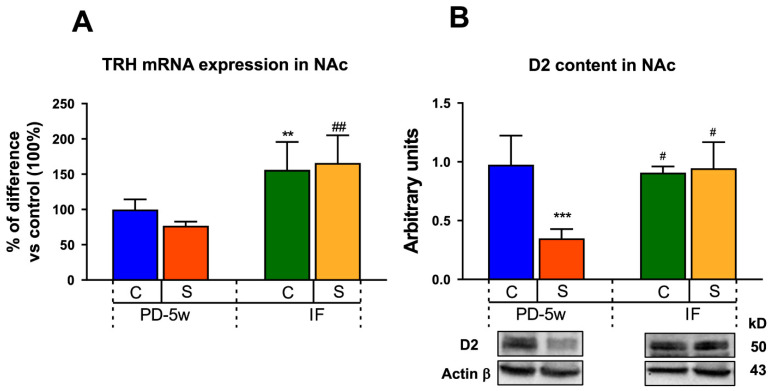
Effect of stress and intermittent fasting on limbic parameters. (**A**) TRH mRNA expression and (**B**) dopamine receptor type 2 (D2) content in the nucleus accumbens (NAc) of the control or stressed animals fed with a PD and subjected to two feeding schedules: food available all day (with a cumulative intake time of 5 weeks, C-PD-5w, S-PD-5w) or intermittent fasting (C-IF, S-IF) for 3 additional weeks. Data are expressed as the mean ± SEM expressed in % of difference vs. C-PD-5w, or in arbitrary units of the intensity of D2/actin signals (n = 4 rats/group). ** *p* < 0.01, *** *p* < 0.001 vs. C-PD-5w; ^#^ *p* < 0.05, ^##^ *p* < 0.01 vs. S-PD-5w.

When analyzing the NAc TRH mRNA expression in both the C-IF and the S-IF animals, we observed an increment when compared to their respective all-day-fed controls (C-PD-5w and S-PD-5w), (Figure 7A). Two-way ANOVA showed that the feeding schedule had a significant effect of F_(1,14)_ = 5.80, *p* < 0.05.

When comparing the accumbal D2 expression, we observed that only the S-PD-5w group showed a decrease in their D2 density vs. C-PD-5w, which was reversed by the IF schedule (Figure 7B). Two-way ANOVA showed a significant effect of stress of F_(1,9)_ = 5.36, *p* < 0.05 and of the interaction between stress and feeding schedule of F_(1,9)_ = 6.5, *p* < 0.05.

We also found that the duration of eating palatable food was a relevant factor for D2 expression changes. After 5 weeks of PD intake, the D2 density in NAc decreased to 39% and 14% vs. 2 weeks in the stressed or non-stressed animals, respectively, (C-PD-2w: 100%; 2.5 ± 0.2 a.u.). Furthermore, after 5 weeks, S-PD-5w showed a reduction of 64% in D2 density vs. C-PD-5w (1 ± 0.2 a.u.), supporting an additive effect of stress and palatable food only in the long term (Figure 7B). There was a significant effect of stress of F_(1,19)_ = 11.19, *p* < 0.01, feeding schedule of F_(2,19)_ = 19.58, *p* < 0.001, and of the interaction between stress and feeding schedule of F_(2,19)_ = 6.58, *p* < 0.01.

## 4. Discussion

In both rodents and humans, chronic stress is characterized by a sustained elevation of serum CORT levels, which is a steroid hormone that is implicated in different metabolic, neurologic, and behavioral changes as adaptive processes that allow individuals to cope with a persistent challenge. High serum CORT levels are responsible for the negative emotions that are experienced by individuals, including anxiety, irritability, and impulsivity, among others, which trigger some compensatory behaviors that ameliorate the adverse perception of stressful situations, such as a high intake of palatable food.

### 4.1. Two-Week Stressed and Non-Stressed Rats Eating Chow or Palatable Food

The increased intake of palatable food in the stress-exposed rats allowed the animals to restore the energy stores that were depleted by CORT actions, which were elevated by the isolation. In contrast, when the stressed animals were fed with chow pellets, they did not show any change in their food intake, which supported the specificity of the high-fat-high-sugar palatable food to activate the rewarding processes in the brain and to motivate the animals to maintain hyperphagia of the PD. Our results are in agreement with the stress-induced high intake of palatable food after 2 weeks of isolation [2,18,19]. The higher intake of the PD, and of fat in particular, has been described as being able to stimulate the activity of the tyrosine hydroxylase enzyme, thus increasing the synthesis of dopamine in the ventral tegmental area (VTA), as well as its release in the NAc. Given that a high-fat-high-sugar diet modifies D1 and D2 dopamine receptors’ density in the NAc, it might enhance the rewarding effects of palatable foods [25].

As previously mentioned, the high palatable food consumption of the S-PD-2w group is known to be a compensatory behavior in response to the stress exposure and elevation of CORT serum levels, which our laboratory study [2] and others [18,19] have observed in single-housed rats. Stressful conditions can impair whole-body energy homeostasis by interfering with the central and peripheral circuits that regulate food intake and energy expenditure, leading to b.w. gain [26]. Adipose tissue is a pivotal organ regulating short- and long-term energy homeostasis in the body. In periods of increased food intake, adipose tissue expands in order to allocate energy excess as triacylglycerides in the lipid vacuole of adipocytes. Healthy adipose tissue expansion results from the coordinated process of hyperplasia (an increase in adipocyte number) and hypertrophy (an increase in adipocyte size) [27]. Hyperplastic adipose tissue growth also favors the differentiation and the recruitment of beige adipocytes, which are abundant in mitochondria as well as in the expression of the electron-transport uncoupler UCP1. These adipocytes dissipate the stored energy as heat, counteracting fat mass accretion [28]. However, a sustained exposure to elevated GC levels may impair adipose tissue hyperplasia and beige adipocyte recruitment [29,30]. Accordingly, the S-PD-2w rats with an early enhancement of CORT serum levels exhibited a significant increase in adipocyte size, denoting hypertrophic adipose tissue and visceral fat accumulation [31]. Thus, 2 weeks of isolation stress was able to impair healthy adipose tissue expansion, despite not being reflected in a significant b.w. gain. S-PD-2w could be experiencing greater muscle wasting than the controls due to an increase in the local T_3_ concentration by a higher activity of the muscular deiodinase enzyme, which has an inverse relation with CORT levels [32], however, this awaits confirmation.

Stress also reduced the number of thermogenic beige adipocytes in S-PD-2w, hampering energy expenditure and b.w. regulation. This was associated with a decrease in the expression of the coactivator PGC1α, which is a critical component of the transcriptional machinery that activates thermogenic gene expression in beige adipocytes, including that of UCP1 [33,34]. The initial increase in GCs in this group might impair PGC1α expression and activity, preventing the transdifferentiation of white to beige adipocytes, as has been described [35]. Overall, the detrimental effects of CORT on adipose tissue differentiation and thermogenesis might affect the energy metabolism and ultimately promote obesity.

#### 4.1.1. Neuroendocrine Changes

The TRH mRNA levels decreased in the PVN of the stressed chow-fed rats (S-CD), a congruent change with the repressive effect of CORT on TRH transcription, since the pro-TRH gene promoter contains a consensus site for the glucocorticoid receptor (GR) [24,36]. This observation supported the idea that PVN TRH expression is downstream of the CORT endocrine effects in the isolation-stress group, which might induce a hypothyroideal condition, as has been previously described [7].

Interestingly, in the S-PD-2w rats, the TRH mRNA levels did not decrease, supporting the idea that the ingestion of palatable food decreased CORT levels and, in consequence, maintained TRH expression in basal levels. A high-fat diet intake reduces the stress-induced high serum levels of CORT [37,38], as well as animals’ negative emotions that are associated with the exposure to a threatful challenge. This is well described in chronically isolated stressed male rats during 2 weeks with access to chocolate milk, which showed basal levels of serum GCs, in contrast to the high CORT levels that were exhibited by the stressed rats that only eat chow food [2]. Our present findings support those observations, given that after 15 days of isolation the CORT levels decreased in the S-PD-2w rats to basal values, but those of the chow-fed rats (S-CD-2w) remained elevated. Our results also have suggested that GCs participated in the induction of palatable food hyperphagia, since once the GCs had triggered the activation of the PD intake, the motivation for the high-fat-high-sugar food was sustained, even after the reduction in GC serum content.

#### 4.1.2. Limbic Changes

Our results regarding TRH expression in the NAc are important to support the putative role of the peptide in regulating the stress-induced high intake of palatable food. The NAc is involved in controlling the motivated behaviors that are reinforced and repeated by individuals as a response to the pleasurable characteristics of the reward, which, in this case, is represented by the high-fat and high-sugar content of the chocolate milk. A decreased TRH expression in the NAc has been associated with a higher intake of palatable food in chronically stressed rats [2]. Moreover, elevated CORT levels modulate the accumbal TRHergic pathway’s involvement in palatable food hyperphagia, since the blockade of GR in the NAc avoids both the decrease in the TRH mRNA levels in this region and the high palatable food ingestion of the stressed rats. In this study we corroborated the participation of the accumbal TRHergic system in the regulation of stress-induced hedonic feeding, since the peptide’s expression diminished in the NAc of the stressed animals when consuming a high volume of palatable food (S-PD-2w). The effect of stress in inducing hyperphagia was specific for the PD, since the stressed animals that were fed with a chow diet (S-CD-2w) also displayed a reduction in TRH mRNA levels in the NAc, but did not ingest a higher amount of chow than C-CD-2w. Overall, it appears that the GC levels downregulated TRH synthesis in the NAc, and the low expression of TRH in this brain region might be involved only in the stress-induced hyperphagia of palatable food in animals, but not of chow food. As expected, we found that stress and palatable food intake for 2 weeks induced D2 receptor density reduction in the NAc. It is proposed that a previous high dopamine release induces the desensitization of the D2 receptor in NAc neurons as a compensatory mechanism for the hyperactivity of the dopaminergic pathway after animals are presented with a rewarding stimulus, such as palatable food [39]. A decreased density of D2 is found in the striatal brain region of obese patients [40] and of stressed patients [41], as well as in obese rodents [42]. Since we did not find an additive effect of stress and a PD, it is likely that both of these factors affected the same pathway to modify D2 density in the NAc. Furthermore, after 2 weeks of isolation, the PD intake, or a combination of both conditions, induced a decrease in the accumbal D2 content to similar levels; however, it was inversely correlated only with the hyperphagia of palatable food but not with chow food intake.

### 4.2. Effects of 3 Weeks of Intermittent Fasting Schedule in Stressed and Non-Stressed Rats Fed with Palatable Food

Stress-exposed rats with access to palatable food (S-PD-5w) did not show a higher chocolate milk intake than the controls, as observed after 2 weeks. This might result from the CORT serum levels that were found in basal values in both of the groups, which might have been due to the palatable food ingestion [37,38]. As a consequence, their b.w. gain was also similar. However, the adipocyte size was higher, and the number of thermogenic beige cells decreased in S-PD-5w, as well as their expression of UCP1 and PGC1α, suggesting that the early elevation of CORT in the stress-exposed animals triggered alterations in the adipose tissue metabolism that persisted in the long term.

Interestingly, an IF regimen was able to reduce the stress-induced high intake of chocolate milk and to increase chow consumption in both of the IF groups vs. the all-day-fed animals. As the IF groups were allowed to eat only during 8 h of their activity phase, our results showed that subjecting the rats to IF was sufficient to reduce the intake of the PD, even if it was offered *ad libitum*. The b.w. of the IF animals also decreased as a result of the lower food intake, along with an increased number of beige adipose cells. These results uphold this feeding schedule as a successful non-pharmacologic strategy for the treatment of overweight individuals and obesity.

To explain the effect of IF on palatable food intake, we deemed it possible that the circadian-regulated expression of the brain feeding signals in the IF groups was switched-on and efficiently coordinated with the light-induced peripheral anabolic hormones’ release, in such a way that the appetite-stimulating system was turned off for a longer period (16 h of fasting per day). It is also plausible that, as the NAc is now considered as a region where the expression of the dopaminergic system’s genes are synchronized with circadian rhythms [43], offering food to animals only at the fixed schedule during their active phase might be working as a zeitgeber that entrained the accumbal reward’s signals to motivate the animals to eat only during that period, and to inhibit feeding during their resting phase. The effect of the activation of the reward system only during the activity phase, as induced by our IF schedule, has also proven to be beneficial in ameliorating the consumption of drugs of abuse induced by chronodisruption of the reward system [44].

The entraining of the circadian regulation of the reward system’s genes to the fixed feeding schedule seemed to avoid the dopaminergic system regulation of the rewarding process of the palatable food in the stressed animals (S-IF). The chronic intake of saturated fats reduces the density of D2 in the NAc, which impedes the released dopamine to stimulate its receptors and to induce the satisfactory experience of the rewarding effects of fat [39]. Here, IF seemed to avoid the decrease in the accumbal D2 density, even when a high-fat-high-sugar food was consumed by the stressed animals (S-IF). A reduced D2 density in the NAc of obese people supports a low perception of food-rewarding properties that is related to their hyperphagia [40].

The rats that were subjected to the IF schedule reduced their b.w. compared to the groups with constant food availability, independently of stress exposure. This phenomenon could be partially due to increased adipose tissue thermogenesis, as evidenced by the high expression levels of PGC1α and UCP-1 proteins and beige cell abundance in the adipose tissue of the rats that were subjected to IF. These changes, along with the reduction in adipocyte size, suggested that IF can stimulate thermogenic beige adipocyte proliferation and activity, increasing energy expenditure and leading to a NEB that favors body weight reduction.

This is similar to that observed in rats that were fed with a chow or high-fat diet and subjected to an IF schedule, which exhibited an increased content of UCP1 and PGC1α, as well as more browning of visceral adipose tissue [45,46,47]. Moreover, the higher T_3_ serum concentration that is shown in IF rats correlated with their increased adipocytes’ UCP1 and PGC1α mRNAs, given that their gene promoters have a site for TH binding (thyroid hormones’ response element, TRE) [48,49]. The smaller size of the adipocytes exhibited by C-IF and S-IF groups could also be related to a higher activity of the lipoprotein lipase enzyme, as it has been described in mesenteric, perirenal, and subcutaneous adipose tissue of rats that were subjected to time-restricted feeding [50]; however, this needs further analysis. Furthermore, even if the IF rats were not food restricted or chow fed, they voluntarily reduced their chocolate milk drinking and increased the ingestion of chow food vs. the all-day-fed groups. Thus, the reduction in b.w. gain of the IF animals seemed to be in part due to their lower energy intake.

#### 4.2.1. Neuroendocrine Changes

Given the anorexic effects of TRH administration [51,52,53,54], we expected to observe high mRNA levels in the PVN of both of the IF groups, which ingested less palatable food than those that were maintained in an all-day-fed schedule; however, this was only true for the stressed-IF animals. The TRHergic neurons of the PVN receive different inputs, such as ARC NPY-expressing afferents, which send their projections to the PVN and are able to decrease the TRH mRNA levels and the HPT axis function [55,56]. Our results support the idea that the high TRH expression in the PVN might be a consequence of the reduced NPY levels that impaired the NPY orexigenic effects in IF-stressed animals, even when they were offered palatable food. The factor that was involved in inducing those changes in S-IF and not in C-IF could be a greater sensitivity to leptin and the return to basal levels of GCs (after being elevated before subjection to a PD and IF schedule), which allowed the NPY expression to decrease in S-IF only.

The S-PD-5w rats exhibited CORT basal levels, likely due to the ingestion of a PD. In addition, even when C-IF and S-IF ate a lower amount of food than their all-day-eating controls, they did not show any elevation in their CORT levels either, as seen in the NEB conditions [7]. This suggests that IF is a feeding schedule that avoids the activation of the adrenal axis in stressed rats even when it decreases their food intake.

The increased T_3_ serum levels that were observed in C-IF and S-IF animals might be responsible for their low b.w. gain, which was associated with their increased HPT axis activity that stimulated their metabolic rate. Only S-IF showed high levels of T_3_ along with increased PVN TRH mRNA expression, which revealed that the negative feedback of the TH to the hypothalamic cells was blocked, allowing the animals to metabolize the lipids and the carbohydrates of the chocolate milk efficiently. In contrast, although T_3_ was also elevated in the C-IF group, the PVN TRH mRNA levels did not decrease, but were maintained at control levels. This might slow the lipid degradation and b.w. loss of that group in the long term. The decrease in total energy intake is known to induce a negative energy balance, which is represented by a decreased expression of TRH in the PVN and low T_3_ serum levels in order to preserve the energy reserves. However, we found an increase in the T_3_ serum concentration in the C-IF and S-IF groups that was not able to reduce PVN TRH transcription. Therefore, IF allowed a reduction in food intake with no adaptations of the HPT axis, due to the NEB.

#### 4.2.2. Limbic Changes

The IF schedule was able to increase the TRH mRNA in the NAc of the stressed and non-stressed animals (C-IF and S-IF), which was associated with their reduced ingestion of palatable food. In contrast, the animals with all-day access to food showed no changes in TRH expression and greater food intake and b.w. As mentioned previously, the NAc expression of the TRH decreases in chronically stressed animals, which correlates with their higher intake of palatable food. Furthermore, the injection of the GR antagonist into the NAc reduces the hyperphagic behavior of rats along with enhancing the TRH mRNA levels in this region [2]. Therefore, our results here supported the anorexigenic role of the accumbal TRH pathway, but also that IF was able to dampen both the effects of the PD and of stress for inducing hyperphagia through activating the TRHergic system of the NAc.

After 5 weeks of PD intake, both the stressed and the non-stressed groups showed a greater decrease in accumbal D2 receptor density than those of their 2-week respective groups, which supports the idea that D2 is sensitive to a sustained ingestion of energy-rich food. This is in agreement with the findings showing that the consumption of a PD stimulates the dopaminergic pathway [57,58,59] and decreases D2 density [39]. Furthermore, after 5 weeks, the stressed group with a PD (S-PD-5w) showed decreased accumbal D2 content vs. the non-stressed group (C-PD-5w); these results suggest that there was an additive effect of stress and PD in changing the D2 protein expression, but only at longer periods of ingesting chocolate milk.

The IF schedule reversed the reduction in D2 in the stressed animals. As both of the groups that were subjected to IF decreased their palatable food intake in comparison to the all-day-eating rats, the reduced D2 content in the NAc could not be associated with the low PD intake that was observed. In contrast, this might be related to the low b.w. gain that was observed in C-IF and S-IF vs. their respective controls (C-PD-5w and S-PD-5w). This association between high b.w. and low D2 density in the NAc has been proposed previously, since rodents eating an equal amount of Kcal from a high-fat or a control diet showed decreased D2 density in the NAc only in heavier HFD-fed rats [39]. Thus, the b.w. gain seems to be more related to the D2 changes than to PD intake [60], which is likely due to an altered concentration of peripheral adipokines in lighter animals, which have receptors in the NAc or VTA and are able to modify the dopaminergic pathway [61,62].

## 5. Conclusions

Our results have supported the benefits of an IF schedule as a non-pharmacologic strategy to prevent and treat stress-associated obesity, by reducing the stress-induced hyperphagia of palatable food. The beneficial effects of IF involve the upregulation of the HPT axis function and the downregulation of the adrenal axis, favoring energy expenditure by beige adipocytes thermogenesis, as well as the modulation of the reward system through attenuating the accumbal D2 density decrease and that of TRHergic accumbal function.

## Figures and Tables

**Table 1:** Effect of palatable diet on adipocytes morphology and thermogenesis.

	C-PD-2w	S-PD-2w
Adipocyte size (µm^2^)	1351 ± 27	1855 ± 68 ***
Beige adipocytes (number/field)	1.2 ± 0.3	0.4 ± 0.2 *
UCP1 mRNA (relative abundance)	1.1 ± 0.2	0.5 ± 0.2 *
PGC1α mRNA (relative abundance)	1.1 ± 0.2	0.3 ± 0.1 **

C-PD-2w: non-stressed rats fed with a palatable diet for 2 weeks; S-PD-2w: stressed rats fed with a palatable diet for 2 weeks; UCP1: uncoupling protein 1; PGC1α: PPARƔ coactivator 1α. Data represent the mean ± SEM (n = 3–4 rats/group). * *p* < 0.05 ** *p* < 0.01, *** *p* < 0.001 vs. C-PD-2w.

## Data Availability

The data presented in this study are available on request from the corresponding author.

## References

[1] Falcón E., Valdés-Moreno M., Rodríguez C., Sanabrais-Jiménez M., Hernández-Muñoz S., Camarena B., de Gortari P. (2021). Interaction between three stress-related gene polymorphisms and food addiction increases the risk to develop obesity in a sample of Mexican people attending a nutrition clinic. Psychoneuroendocrinology.

[2] Alvarez-Salas E., González A., Amaya M.I., de Gortari P. (2021). Accumbal TRH is downstream of the effects of isolation stress on hedonic food intake in rats. Nutr. Neurosci..

[3] Hewagalamulage S.D., Lee T.K., Clarke I.J., Henry B.A. (2016). Stress, cortisol, and obesity: A role for cortisol responsiveness in identifying individuals prone to obesity. Domest. Anim. Endocrinol..

[4] Yau Y.H.C., Potenza M.N. (2013). Stress and eating behaviors. Minerva Endocrinol..

[5] van Rossum E.F.C. (2017). Obesity and cortisol: New perspectives on an old theme. Obesity.

[6] Wester V.L., Staufenbiel S.M., Veldhorst M.A.B., Visser J.A., Manenschijn L., Koper J.W., Klessens-Godfroy F.J.M., Van Den Akker E.L.T., Van Rossum E.F.C. (2014). Long-term cortisol levels measured in scalp hair of obese patients. Obesity.

[7] van Haasteren G.A., Rondeel J.M.M., Themmen A.P.N., De Jong F.H., Valentijn K., Vaudry H., Bauer K., Visser T.J., de Greef W.J. (1995). Starvation-induced changes in the hypothalamic content of prothyrotrophin-releasing hormone (proTRH) mRNA and the hypothalamic release of proTRH-derived peptides: Role of the adrenal gland. J. Endocrinol..

[8] Nillni E.A., Vaslet C., Harris M., Hollenberg A., Bjorbaek C., Flier J.S. (2000). Leptin Regulates Prothyrotropin-releasing Hormone Biosynthesis: Evidence for direct and indirect pathways. J. Biol. Chem..

[9] Alkemade A., Unmehopa U.A., Wiersinga W.M., Swaab D.F., Fliers E. (2005). Glucocorticoids Decrease Thyrotropin-Releasing Hormone Messenger Ribonucleic Acid Expression in the Paraventricular Nucleus of the Human Hypothalamus. J. Clin. Endocrinol. Metab..

[10] Kakucska I., Qi Y., Lechan R.M. (1995). Changes in adrenal status affect hypothalamic thyrotropin-releasing hormone gene expression in parallel with corticotropin-releasing hormone. Endocrinology.

[11] Adafer R., Messaadi W., Meddahi M., Patey A., Haderbache A., Bayen S., Messaadi N. (2020). Food Timing, Circadian Rhythm and Chrononutrition: A Systematic Review of Time-Restricted Eating’ s Effects on Human Health. Nutrients.

[12] Morales-Suarez-Varela M., Collado Sánchez E., Peraita-Costa I., Llopis-Morales A., Soriano J.M., Fiorentino V. (2021). Intermittent Fasting and the Possible Benefits in Obesity, Diabetes, and Multiple Sclerosis: A Systematic Review of Randomized Clinical Trials. Nutrients.

[13] Zubrzycki A., Cierpka-Kmiec K., Kmiec Z., Wronska A. (2018). The role of low-calorie diets and intermittent fasting in the treatment of obesity and type-2 diabetes. J. Physiol. Pharmacol..

[14] Kim B.H., Joo Y., Kim M.S., Choe H.K., Tong Q., Kwon O. (2021). Effects of Intermittent Fasting on the Circulating Levels and Circadian Rhythms of Hormones. Endocrinol. Metab..

[15] García-Luna C., Soberanes-Chávez P., de Gortari P. (2017). Prepuberal light phase feeding induces neuroendocrine alterations in adult rats. Mol. Neurophysiol. Lab..

[16] Longo V.D., Panda S., Adamello V., Jolla L. (2016). Fasting, circadian rhythms, and time restricted feeding in healthy lifespan. Cell Metab..

[17] Hatori M., Vollmers C., Zarrinpar A., Ditacchio L., Eric A., Gill S., Leblanc M., Chaix A., Joens M., James A. (2013). Time restricted feeding without reducing caloric intake prevents metabolic diseases in mice fed a high fat diet. Cell Metab..

[18] Nakhate K., Subhedar N. (2010). Central regulation of feeding behavior during social isolation of rat: Evidence for the role of endogenous CART system Drug design and its Pharmacological Screening View project Ventral tegmental area View project. Artic. Int. J. Obes..

[19] Krolow R., Noschang C., Arcego D.M., Huffell A.P., Marcolin M.L., Benitz A.N., Lampert C., Fitarelli R.D., Dalmaz C. (2013). Sex-specific effects of isolation stress and consumption of palatable diet during the prepubertal period on metabolic parameters. Metabolism.

[20] Paxinos G., Watson C. (2005). The Rat Brain in Stereotaxic Coordinates.

[21] Chomczynski P., Sacchi N. (1987). Single-step method of RNA isolation by acid guanidinium thiocyanate-phenol-chloroform extraction. Anal. Biochem..

[22] Galarraga M., Campión J., Munõz-Barrutia A., Boqué N., Moreno H., Martínez J.A., Milagro F., Ortiz-de-Solórzano C. (2012). Adiposoft: Automated software for the analysis of white adipose tissue cellularity in histological sections. J. Lipid Res..

[23] Delgadillo-Puga C., Noriega L.G., Morales-Romero A.M., Nieto-Camacho A., Granados-Portillo O., Rodríguez-López L.A., Alemán G., Furuzawa-Carballeda J., Tovar A.R., Cisneros-Zevallos L. (2020). Goat’s Milk Intake Prevents Obesity, Hepatic Steatosis and Insulin Resistance in Mice Fed A High-Fat Diet by Reducing Inflammatory Markers and Increasing Energy Expenditure and Mitochondrial Content in Skeletal Muscle. Int. J. Mol. Sci..

[24] Kakucska I., Romero L.I., Clark B.D., Rondeel J.M.M., Qi Y., Alex S., Emerson C.H., Lechan R.M. (1994). Suppression of thyrotropin-releasing hormone gene expression by interleukin-1-beta in the rat: Implications for nonthyroidal illness. Neuroendocrinology.

[25] Alsio J., Olszewski P.K., Norbäck A.H., Gunnarsson Z.E.A., Levine A.S., Pickering C., Schiöth H.B. (2010). Dopamine D1 receptor gene expression decreases in the nucleus accumbens upon long-term exposure to palatable food and differs depending on diet-induced obesity phenotype in rats. Neuroscience.

[26] Srivastav P., Broadbent S., Vaishali K., Nayak B., Bhat H.V. (2020). Prevention of adolescent obesity: The global picture and an indian perspective. Diabetes Metab. Syndr..

[27] Porro S., Genchi V.A., Cignarelli A., Natalicchio A., Laviola L., Giorgino F., Perrini S. (2020). Dysmetabolic adipose tissue in obesity: Morphological and functional characteristics of adipose stem cells and mature adipocytes in healthy and unhealthy obese subjects. J. Endocrinol. Investig..

[28] Rabiee A. (2020). Beige Fat Maintenance; Toward a Sustained Metabolic Health. Front. Endocrinol..

[29] Zubiría M.G., Alzamendi A., Moreno G., Portales A., Castrogiovanni D., Spinedi E., Giovambattista A. (2016). Relationship between the Balance of Hypertrophic/Hyperplastic Adipose Tissue Expansion and the Metabolic Profile in a High Glucocorticoids Model. Nutrients.

[30] Lv Y.F., Yu J., Sheng Y.L., Huang M., Kong X.C., Di W.J., Liu J., Zhou H., Liang H., Ding G.X. (2018). Glucocorticoids Suppress the Browning of Adipose Tissue via miR-19b in Male Mice. Endocrinology.

[31] Rosmond R., Dallman M.F., Björntorp P. (1998). Stress-Related Cortisol Secretion in Men: Relationships with Abdominal Obesity and Endocrine, Metabolic and Hemodynamic Abnormalities. J. Clin. Endocrinol. Metab..

[32] Cavalieri R.R., Castle J.N., McMahon F.A. (1984). Effects of Dexamethasone on Kinetics and Distribution of Triiodothyronine in the Rat. Endocrinology.

[33] Frontini A., Giordano A., Cinti S. (2012). Endothelial cells of adipose tissues: A niche of adipogenesis. Cell Cycle.

[34] Sidossis L., Kajimura S. (2015). Brown and beige fat in humans: Thermogenic adipocytes that control energy and glucose homeostasis. J. Clin. Investig..

[35] Rahnert J.A., Zheng B., Hudson M.B., Woodworth-Hobbs M.E., Price S.R. (2016). Glucocorticoids Alter CRTC-CREB Signaling in Muscle Cells: Impact on PGC-1α Expression and Atrophy Markers. PLoS ONE.

[36] Lee S.L., Stewart K., Goodman R.H. (1988). Structure of the gene encoding rat thyrotropin releasing hormone. J. Biol. Chem..

[37] Pecoraro N., Reyes F., Gomez F., Bhargava A., Dallman M.F. (2004). Chronic Stress Promotes Palatable Feeding, which Reduces Signs of Stress: Feedforward and Feedback Effects of Chronic Stress. Endocrinology.

[38] Ortolani D., Oyama L.M., Ferrari E.M., Melo L.L., Spadari-Bratfisch R.C. (2011). Effects of comfort food on food intake, anxiety-like behavior and the stress response in rats. Physiol. Behav..

[39] Johnson P.M., Kenny P.J. (2010). Dopamine D2 receptors in addiction-like reward dysfunction and compulsive eating in obese rats. Nat. Neurosci..

[40] Wang G., Volkow N.D., Logan J., Pappas N.R., Wong C.T., Zhu W., Netusil N. (2001). Brain dopamine and obesity. Lancet.

[41] Volkow N.D., Wang G.J., Maynard L., Jayne M., Fowler J.S., Zhu W., Logan J., John Gatley S., Ding Y.S., Wong C. (2003). Brain dopamine is associated with eating behaviors in humans. Int. J. Eat. Disord..

[42] Hamdi A., Porter J., Chandan P. (1992). Decreased striatal D2 dopamine receptors in obese Zucker rats: Changes during aging. Brain Res..

[43] Becker-Krail D.D., Walker W.H., Nelson R.J. (2022). The Ventral Tegmental Area and Nucleus Accumbens as Circadian Oscillators: Implications for Drug Abuse and Substance Use Disorders. Front. Physiol..

[44] Tamura E.K., Oliveira-Silva K.S., Ferreira-Moraes F.A., Marinho E.A.V., Guerrero-Vargas N.N. (2021). Circadian rhythms and substance use disorders: A bidirectional relationship. Pharmacol. Biochem. Behav..

[45] Aouichat S., Chayah M., Bouguerra-Aouichat S., Agil A. (2020). Time-Restricted Feeding Improves Body Weight Gain, Lipid Profiles, and Atherogenic Indices in Cafeteria-Diet-Fed Rats: Role of Browning of Inguinal White Adipose Tissue. Nutrients.

[46] Liu B., Page A.J., Hutchison A.T., Wittert G.A., Heilbronn L.K. (2019). Intermittent fasting increases energy expenditure and promotes adipose tissue browning in mice. Nutrition.

[47] Li G., Xie C., Lu S., Nichols R.G., Tian Y., Li L., Patel D., Ma Y., Brocker C.N., Yan T. (2017). Intermittent Fasting Promotes White Adipose Browning and Decreases Obesity by Shaping the Gut Microbiota. Cell Metab..

[48] Lanni A., Moreno M., Lombardi A., Goglia F. (2003). Thyroid hormone and uncoupling proteins. FEBS Lett..

[49] Wulf A., Harneit A., Kröger M., Kebenko M., Wetzel M.G., Weitzel J.M. (2008). T3-mediated expression of PGC-1α via a far upstream located thyroid hormone response element. Mol. Cell. Endocrinol..

[50] Sugden M.C., Grimshaw R.M., Lall H., Holness M.J. (1994). Regional variations in metabolic responses of white adipose tissue to food restriction. Am. J. Physiol. Endocrinol. Metab..

[51] Puga L., Alcántara-Alonso V., Coffeen U., Jaimes O., de Gortari P. (2016). TRH injected into the nucleus accumbens shell releases dopamine and reduces feeding motivation in rats. Behav. Brain Res..

[52] Vijayan E., McCann S.M. (1977). Suppression of feeding and drinking activity in rats following intraventricular injection of thyrotropin releasing hormone (TRH) 1. Endocrinology.

[53] Suzuki T., Kohno H., Sakurada T., Tadano T., Kisara K. (1982). Intracranial injection of thyrotropin releasing hormone (TRH) suppresses starvation-induced feeding and drinking in rats. Pharmacol. Biochem. Behav..

[54] Choi Y.H., Hartzell D., Azain M.J., Baile C.A. (2002). TRH decreases food intake and increases water intake and body temperature in rats. Physiol. Behav..

[55] Fekete C., Sarkar S., Rand W.M., Harney J.W., Emerson C.H., Bianco A.C., Beck-Sickinger A., Lechan R.M. (2002). Neuropeptide Y1 and Y5 Receptors Mediate the Effects of Neuropeptide Y on the Hypothalamic-Pituitary-Thyroid Axis. Endocrinology.

[56] Fekete C., Kelly J., Mihály E., Sarkar S., Rand W.M., Légrádi G., Emerson C.H., Lechan R.M. (2001). Neuropeptide Y Has a Central Inhibitory Action on the Hypothalamic-Pituitary-Thyroid Axis. Endocrinology.

[57] Bassareo V., Di Chiara G. (1997). Differential Influence of Associative and Nonassociative Learning Mechanisms on the Responsiveness of Prefrontal and Accumbal Dopamine Transmission to Food Stimuli in Rats Fed Ad Libitum. J. Neurosci..

[58] Hernandez L., Hoebel B.G. (1988). Feeding and hypothalamic stimulation increase dopamine turnover in the accumbens. Physiol. Behav..

[59] Roitman M.F., Stuber G.D., Phillips P.E.M., Wightman R.M., Carelli R.M. (2004). Dopamine Operates as a Subsecond Modulator of Food Seeking. J. Neurosci..

[60] Stice E., Yokum S., Blum K., Bohon C. (2010). Weight Gain Is Associated with Reduced Striatal Response to Palatable Food. J. Neurosci..

[61] Hommel J.D., Trinko R., Sears R.M., Georgescu D., Liu Z.W., Gao X.B., Thurmon J.J., Marinelli M., DiLeone R.J. (2006). Leptin Receptor Signaling in Midbrain Dopamine Neurons Regulates Feeding. Neuron.

[62] Fulton S., Pissios P., Manchon R.P., Stiles L., Frank L., Pothos E.N., Maratos-Flier E., Flier J.S. (2006). Leptin Regulation of the Mesoaccumbens Dopamine Pathway. Neuron.

